# The homogeneous mutation status of a 22 gene panel justifies the use of serial sections of colorectal cancer tissue for external quality assessment

**DOI:** 10.1007/s00428-015-1789-5

**Published:** 2015-06-06

**Authors:** Jeroen R. Dijkstra, Bastiaan B. J. Tops, Iris D. Nagtegaal, J. Han J. M. van Krieken, Marjolijn J. L. Ligtenberg

**Affiliations:** Department of Pathology, Radboud University Medical Center, PO Box 9101, 6500 HB Nijmegen, The Netherlands; Department of Human Genetics, Radboud University Medical Center, Nijmegen, The Netherlands

**Keywords:** Colon, RAS, Reproducibility, Mutant allele frequency

## Abstract

Testing for treatment related biomarkers in clinical care, like *Ras* mutation status in colorectal cancer (CRC), has increased drastically over recent years. Reliable testing of these markers is pivotal for optimal treatment of patients. Participation in external quality assessment (EQA) programs is an important element in quality management and often obligatory to comply with regulations or for accreditation. Formalin-fixed paraffin-embedded (FFPE) clinical specimens would ideally form the basis for these assessments, as they represent the most common starting material for molecular testing. However, molecular heterogeneity of a lesion in a FFPE tissue block could potentially affect test results of participating laboratories, which might compromise reliability of the quality assessment results. To assess the actual impact of this potential problem, we determined the mutation status of 22 genes commonly mutated in colon cancer in four levels covering 360 μm of 30 FFPE tissue blocks, by Next Generation Sequencing. In each block, the genotype of these genes was identical at all four levels, with only little variation in mutation load. This result shows that the mutation status of the selected 22 genes in CRC specimens is homogeneous within a 360 μm segment of the tumor. These data justify the use of serial sections, within a defined segment of a CRC tissue block, for external quality assessment of mutation analysis.

**Electronic supplementary material:**

The online version of this article (doi:10.1007/s00428-015-1789-5) contains supplementary material, which is available to authorized users.

## Introduction

In recent years, testing of tumor-specific molecular biomarkers to predict the putative response to targeted therapeutics has increased rapidly. An example is the incorporation in standard care of the mutation status of *KRAS* and *NRAS*, which is decisive in starting epidermal growth factor receptor (EGFR)-targeted therapy on metastatic colorectal cancer patients. The European Medicines Agency (EMA) declared that the use of EGFR-targeted therapies should be restricted to patients with a *Ras* wild-type tumor [1, 2]. Mutations in other genes, such as *BRAF* and *PIK3CA*, are now tested in the context of clinical trials and may enter clinical practice in the near future.

Quality assessment is essential to validate the results of these molecular tests, in order to achieve or sustain optimal patient treatment [3]. Furthermore, quality assessment allows comparison of inter-laboratory results, which is pivotal to determine the effectiveness of a treatment regime instigated by the result of a molecular test. To assure high quality of testing, it is essential that each laboratory properly validates its workflow of molecular tests, typically a sequence of techniques [4–7] which for mutation screening consists of DNA extraction, PCR amplification, sequencing, and detection. Test results need to be validated, and participation in external quality assessment (EQA) programs is often obligatory to comply with regulations or a requirement for accreditation as a test provider [8, 9].

Formalin-fixed paraffin-embedded (FFPE) tissue is the most common starting material for molecular testing, independent of the exact workflow or test origin (laboratory developed test or commercially available). Therefore, they provide the ideal basis for validation, EQA programs, and for comparison of method performance. Reference samples need to yield highly reproducible results over the participating laboratories. This is easy to attain with homogeneous liquid samples from which random samples can be drawn but potentially problematic for serial tissue sections from an intrinsically heterogeneous tissue block. Reproducibility problems can arise due to a variety of factors, including variation in the surface area occupied by tumor tissue, proportion of neoplastic cells relative to stromal cells in tumor tissue and/or heterogeneity of molecular abnormalities within a given block of tumor tissue.

Next generation sequencing (NGS) based techniques are rapidly becoming the new standard to evaluate the mutation status of a sample of tumor tissue. They provide highly sensitive semi-quantitative information on all possible mutations in a region of interest of the genome. To assess mutation status of the most commonly mutated genes in colon cancer, a colon-lung cancer panel was designed that allows reliable mutation analysis (on only 10 ng of DNA) of the most frequently mutated regions of the genes for receptor tyrosine kinases *EGFR*, *ERBB2*, *ERBB4*, *MET*, *ALK*, *FGFR1*, *FGFR2*, *FGFR3*, and *DDR2*, their pathway genes *KRAS, NRAS, BRAF, PIK3CA,* and *MAP2K1*, as well as *TP53, STK11, CTNNB1, SMAD4*, *FBXW7,* and *NOTCH1*, on only 10 ng of DNA [10].

We evaluated the reproducibility of genotyping for this panel of tissue sections at different depths of CRC tissue blocks, as this is important for the design of EQA schemes evaluating gene testing for RAS and eventually other genes in the panel, in view of the requirement that serial sections at different depths of a tumor block should generate the same results.

## Materials and methods

### Samples

FFPE tissue samples from resected primary colorectal cancer specimens, stage T3N0M0 or higher and with at least 40 % of neoplastic cells, were obtained from 30 patients who had been treated at the Radboud University Medical Center (RadboudUMC) between 2007 and 2013. Six tumors were microsatellite instable (MSI), 24 microsatellite stable (MSS), and 8 were from patients treated with neoadjuvant chemotherapy. The samples were anonymized for quality assessment purposes, as a result of which they were not subjected to research regulations of RadboudUMC, which require informed consent and permission by the internal review board.

### DNA extraction

**Fig. 1:**
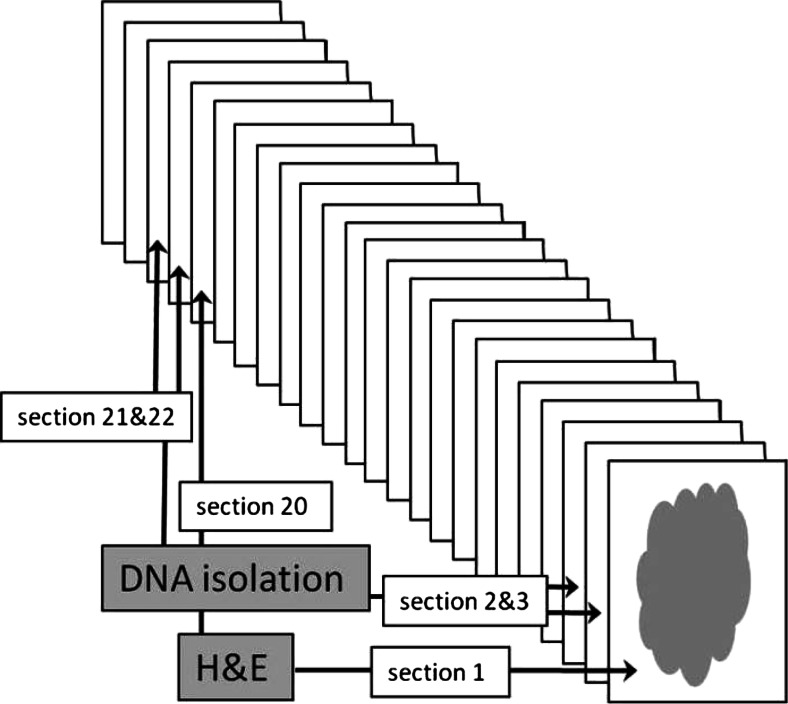
Schematic display of the use of the designated sections. All sections had a thickness of 6 μm

FFPE blocks were cut to obtain 63 consecutive sections of 6 μm. H&E staining was performed on sections 1, 20, 40, and 60. A pathologist assessed each stained section individually and selected the region for macrodissection and estimated the percentage of neoplastic cells. Macrodissected tumor material from two sections following each H&E stained section was pooled for DNA extraction (Fig. 1). Tissue was digested in 110 μl 5 % Chelex-100 resin (Bio-Rad laboratories, Hercules, CA) and 2 mg/ml proteinase K in 10 mM Tris/HCl (pH 8.5), 1 mM EDTA (pH 8.0), 0.01 % Tween-20. Following overnight incubation at 56 °C, the enzyme was heat inactivated at 95 °C for 10 min. After two rounds of centrifugation (16,000 rpm for 1 min) the DNA in the supernatant was quantified using the Qubit platform (Life Technologies, Foster City, CA).

### Sequencing

Bar-coded libraries were prepared from 10 ng DNA using the Ion AmpliSeq Colon and Lung Cancer Panel version 1 (Life Technologies), essentially as described by the manufacturer except for amplification with 23 instead of 21 cycles. Clonal amplification was subsequently performed by emulsion PCR using the One Touch 2 system (Life Technologies). DNA concentration of the resulting libraries was measured using the Qubit platform (Life Technologies). Libraries were quantitatively pooled and run on the Ion Torrent Personal Genome Machine (Life Technologies). Torrent Suite Software v.3.4.2 was used to pre-process raw data. Subsequently, sequence alignment and variant calling were performed using SeqNext software v.4.1.2 (JSI medical systems GmbH, Kippenheim, Germany). Each nucleotide was covered with a minimum of at least 50 reads, and variants were included if they were found at an allelic frequency of at least 10 %.

### Data analysis

Variants were filtered for known SNPs (MAF ≥ 0.02), synonymous mutations, and variants most likely to be false positive. The latter were defined as variants present in most/all of the 120 DNA samples. The remaining mutations were assessed in the Catalogue of Somatic Mutations in Cancer (COSMIC) database [11]. Variants not present in the COSMIC database were subjected to Sanger sequencing to confirm the somatic origin of the specific variant (in normal and tumor tissue).

Differences between the percentages of mutated reads of individual mutations at different levels of a tissue block were evaluated by ANOVA, assuming repeated measurements.

## Results

To assess reproducibility of genotyping within one FFPE colorectal tumor tissue block, 63 sections of 6 μm were cut from 30 different tumors. Sections 1 (level A), 20 (level B), 40 (level C), and 60 (level D) were histologically evaluated (Fig. 1). Of each sample, a comparable region was macrodissected at each level. The estimated percentage of neoplastic cells was similar for the different levels within a block with a maximum of 20 % difference from level A (mean 3.56 %, st dev 5.67) (supplementary data).

Mutation analysis using the Ion AmpliSeq Colon and Lung Cancer Panel version 1 (Life Technologies), was performed on DNA isolated from the macrodissected region of sections 2 and 3 (level A), sections 21, and 22 (level B), sections 41, and 42 (level C) and sections 61 and 62 (level D). Because of poor quality of the DNA from the initial isolation of tumor 18 sections 23 and 24, sections 43 and 44, and section 63 and 64 were used as levels B, C, and D, respectively. Variant calling in *KRAS* (exon 2, 3, and 4), *NRAS* (exon 2 and 3), *BRAF* (exon 15), and *PIK3CA* (exon 10 and 21) was performed on nucleotide coverage of at least 160 reads. The overall lowest mean coverage for these exons was 2396, with a standard deviation of 1449 (Table 1).

**Table 1:** Coverage data of individual fragments of *KRAS*, *NRAS*, *BRAF,* and *PIK3CA*

	*KRAS*			*BRAF*	*NRAS*		*PIK3CA*	
	Exon 2	Exon 3	Exon 4	Exon 15	Exon 2	Exon 3	Exon 10	Exon 21
Min	186	278	256	175	162	160	233	194
Average	3009	4990	3304	2740	3572	3783	4597	3457
St. dev	1505	1963	1750	1449	1395	1396	1585	1376

The number of somatic mutations detected in the different tumors varied from zero to five with a mean of 3.0 in microsatellite instable, 1.4 in microsatellite stable tumors of patients not subjected to neoadjuvant therapy and 1.4 in microsatellite stable tumors of patients subjected to neo-adjuvant therapy (supplementary data). Mutations in *KRAS*, *NRAS*, *BRAF,* and *PIK3CA* were detected in 9, 1, 6, and 5 tumors, respectively (Table 2). The most frequently mutated gene was *TP53*, mutated in 17 tumors. For all mutations the concordance detected at levels A, B, C, and D was 100 %.

**Table 2:** Mean percentage of mutated reads per gene

		Average % mutated reads (st. dev)			
Gene	*n*	Level A	Level B	Level C	Level D
*BRAF*	6	39 (22)	38 (16)	38 (14)	39 (13)
*DDR2*	1	30	40	39	32
*ERBB4*	1	32	31	39	39
*FBXW7*	4	36 (7)	31 (4)	35 (7)	33 (3)
*FGFR3*	1	38	27	25	30
*KRAS*	9	37 (12)	36 (9)	39 (14)	35 (12)
*MAP2K1*	1	24	34	33	21
*NRAS*	1	34	39	39	43
*PIK3CA*	5	26 (4)	25 (6)	27 (4)	26 (6)
*SMAD4*	4	31 (9)	31 (12)	32 (8)	36 (7)
*TP53*	17	47 (17)	43 (15)	45 (16)	47 (16)

**Fig. 2:**
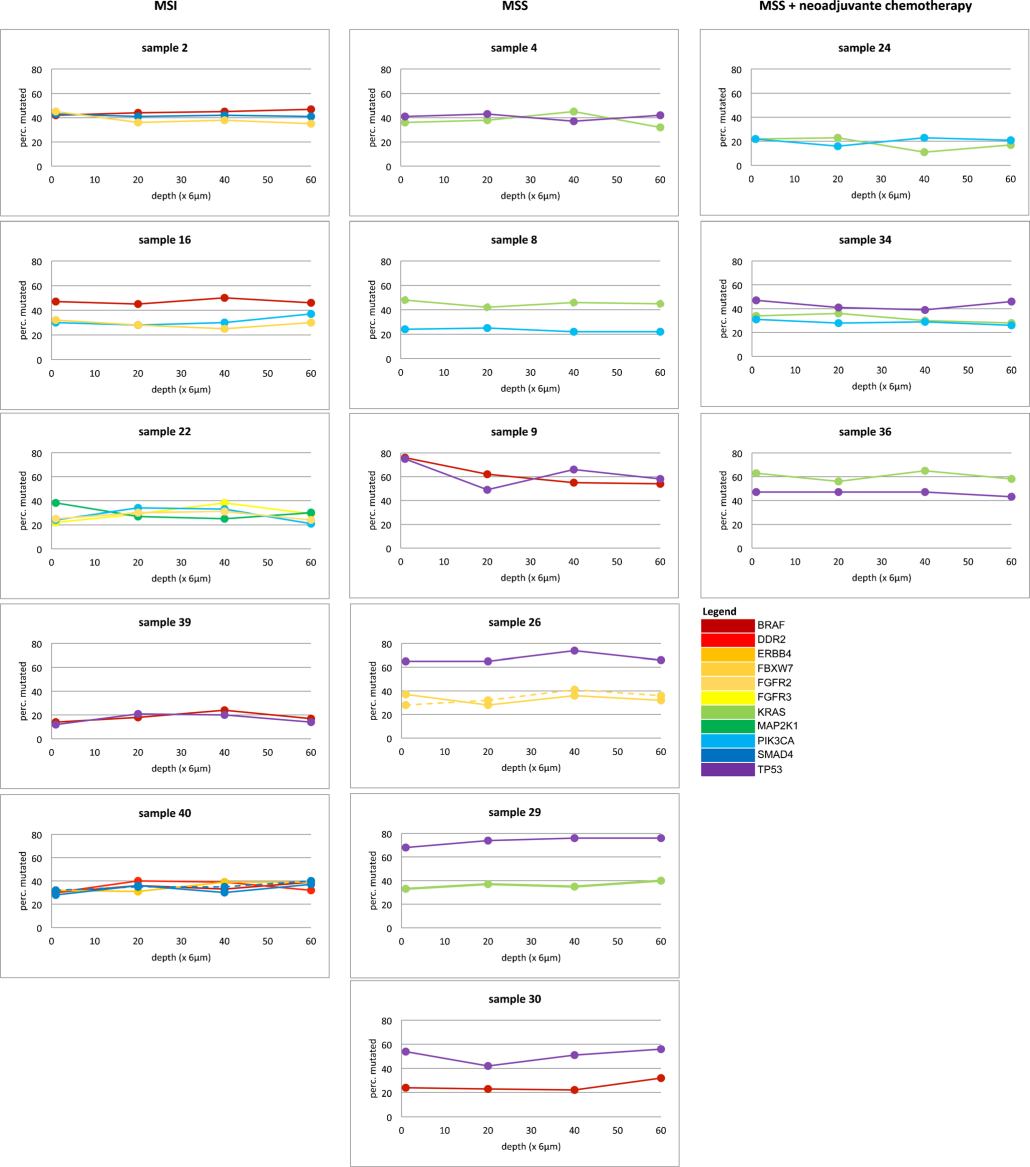
Percentage of mutated reads per mutation for each sample that contains multiple mutations at different levels of the FFPE tissue block (depth). Mutations are represented as color-coded (per gene) continuous lines, except for sample 26 and 40 since in these samples more than one mutation per gene was found

**Fig. 3:**
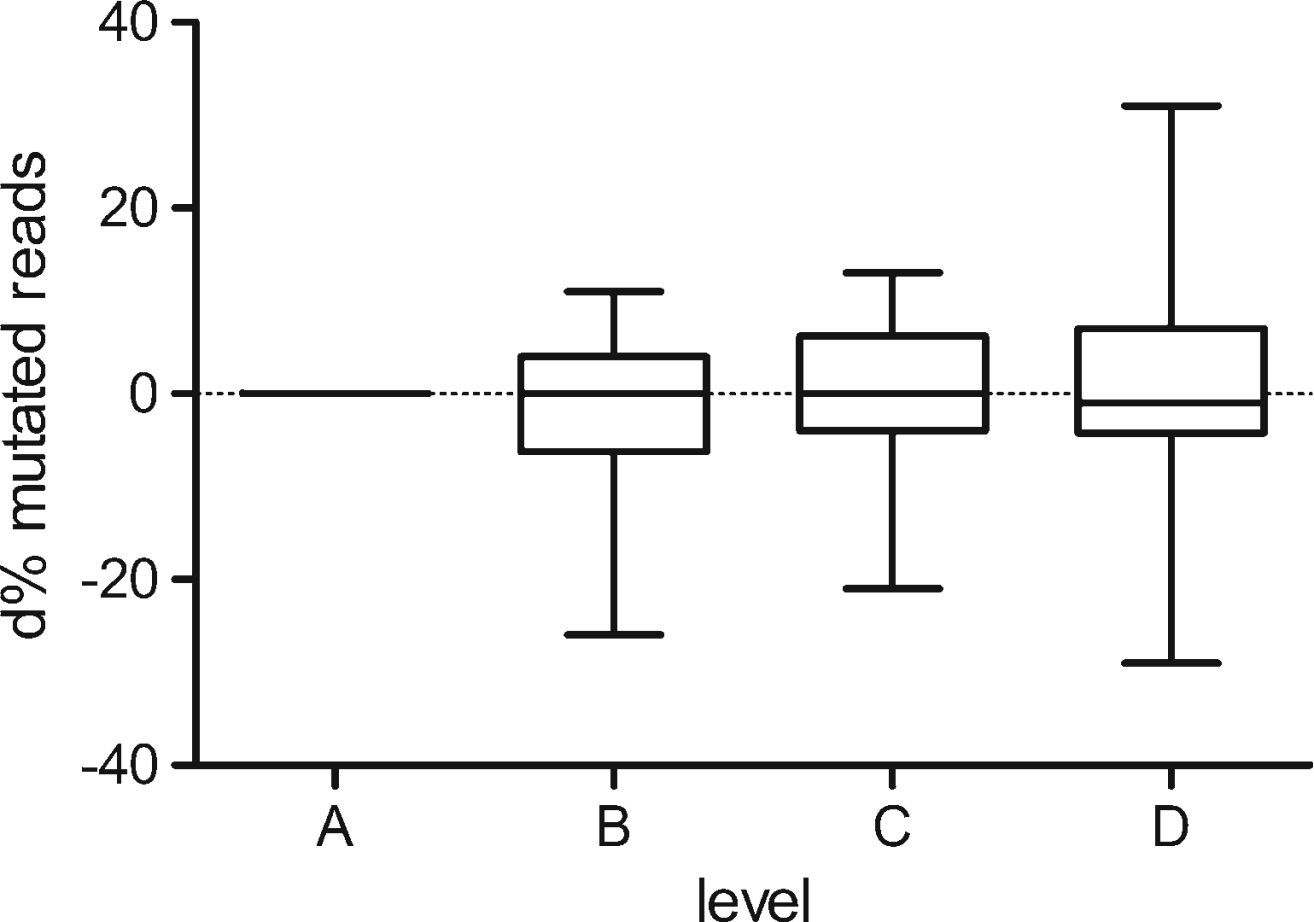
Difference in percentage of mutated reads of all individual mutations at levels B, C, and D in comparison to level A. Difference in percentage of mutant reads at levels B, C, and D compared to that of level A: *p* = 0.392, repeated measures ANOVA

The percentage of variant reads was used to assess the variation in mutation load at the different levels within each tumor block. This varied between different mutations within a given tumor (Fig. 2). However, for a specific mutation the mutation load was stable at all four levels (Fig. 3), as there were no significant differences in percentage of mutant reads between levels A, B, C, and D (p = 0.392). This indicates that NGS is highly reproducible and that heterogeneity between sections at four different levels of tissue blocks is limited.

## Discussion

For each of 30 colorectal tumor blocks tested at four levels separated by 120 μm (hence, a segment of 360 μm between the upper and lower level), the genotype of 22 genes analyzed by next generation sequencing was the same at all four levels. Between different levels of a given tumor, little variation in the percentage of mutant alleles was observed. This shows that NGS-based genetic testing is reproducible and that different levels in a single block of tumor tissue are homogeneous. The results of these analyses justify the use of sections derived from different levels of the same tumor block in current external quality assessment schemes.

For this study, we selected samples with a relatively high percentage of neoplastic cells to facilitate the detection of mutations present in only a subset of neoplastic cells. The mutation load of different mutations in a given tumor differed by a factor two at most, which might reflect subtle copy number changes or copy number neutral loss of heterozygosity events. In four samples (22, 24, 31, and 39), the percentage of mutant alleles was less than 30 % of the percentage of neoplastic cells, which is clearly lower than would be expected if one allele of a given diploid gene would be mutated in all neoplastic cells. This might be due to overestimation of the percentage of neoplastic cells by the pathologist, copy number gains of wild-type alleles, or the presence of these mutations in only a subset of the neoplastic cells. However, as in two (sample 22 and 39) of the three tumors this applies to mutations in multiple genes residing on different chromosomes, overestimation of the percentage of neoplastic cells is the most likely explanation. Therefore, our results do not strongly indicate the existence of tumor heterogeneity for the tested genes in this setting.

In our study, we concentrated on only 30 tumors that were judged eligible for use in quality assessment schemes. This implied morphological homogeneity and avoidance of precancerous regions that might harbor fewer mutations. Differences in mutation load between morphologically different regions within one slide were not examined. Moreover, we studied regions that were at most 360 μm apart, which is far less than the diameter of most tumors. Therefore, our data cannot be used to argue in favor of homogeneity of mutation status throughout an entire primary tumor.

In our next generation sequencing panel, we tested for mutations in genes frequently mutated in colorectal cancer. The panel allows the detection of mutations that are currently considered as actionable (*NRAS*, *KRAS*) or putatively actionable (*BRAF*, *PIK3CA*) in colorectal cancer. The current design of EQA schemes, which distribute sections from different levels of a tissue block and aim at a sensitivity of 10 % mutant alleles, seems therefore also appropriate for these putatively actionable mutations. For reliable detection of mutant alleles present at a lower percentage, other more sensitive approaches would be necessary. However, the clinical relevance of mutations in a low percentage of neoplastic cells is as yet not clear and therefore is not an aim for current EQA schemes.

In conclusion, our data show that the percentage of mutant alleles, of a panel of 22 genes including those that are frequently mutated in colorectal cancer, is similar within a defined depth of a block of colorectal cancer tissue. This justifies the use of serial sections to assess the quality of mutation analyses in different laboratories, as has become customary in EQA schemes.

## Electronic supplementary material

ESM 1 Percentage of mutated reads per sample for each level (A, B, C, and D). Abbreviation: - = no mutation found. (TIFF 295335 kb)
